# ERK and Akt exhibit distinct signaling responses following stimulation by pro-angiogenic factors

**DOI:** 10.1186/s12964-020-00595-w

**Published:** 2020-07-17

**Authors:** Min Song, Stacey D. Finley

**Affiliations:** 1 Department of Biomedical Engineering, University of Southern California, Los Angeles, CA USA; 2 Mork Family Department of Chemical Engineering and Materials Science, University of Southern California, Los Angeles, CA USA; 3 Department of Biological Sciences, University of Southern California, Los Angeles, CA USA

**Keywords:** Computational modeling, Angiogenesis, Growth factor signaling, Sensitivity analysis

## Abstract

**Background:**

Angiogenesis plays an important role in the survival of tissues, as blood vessels provide oxygen and nutrients required by the resident cells. Thus, targeting angiogenesis is a prominent strategy in many different settings, including both tissue engineering and cancer treatment. However, not all of the approaches that modulate angiogenesis lead to successful outcomes. Angiogenesis-based therapies primarily target pro-angiogenic factors such as vascular endothelial growth factor-A (VEGF) or fibroblast growth factor (FGF) in isolation, and there is a limited understanding of how these promoters combine together to stimulate angiogenesis. Targeting one pathway could be insufficient, as alternative pathways may compensate, diminishing the overall effect of the treatment strategy.

**Methods:**

To gain mechanistic insight and identify novel therapeutic strategies, we have developed a detailed mathematical model to quantitatively characterize the crosstalk of FGF and VEGF intracellular signaling. The model focuses on FGF- and VEGF-induced mitogen-activated protein kinase (MAPK) signaling to promote cell proliferation and the phosphatidylinositol 3-kinase/protein kinase B (PI3K/Akt) pathway, which promotes cell survival and migration. We fit the model to published experimental datasets that measure phosphorylated extracellular regulated kinase (pERK) and Akt (pAkt) upon FGF or VEGF stimulation. We validate the model with separate sets of data.

**Results:**

We apply the trained and validated mathematical model to characterize the dynamics of pERK and pAkt in response to the mono- and co-stimulation by FGF and VEGF. The model predicts that for certain ranges of ligand concentrations, the maximum pERK level is more responsive to changes in ligand concentration compared to the maximum pAkt level. Also, the combination of FGF and VEGF indicates a greater effect in increasing the maximum pERK compared to the summation of individual effects, which is not seen for maximum pAkt levels. In addition, our model identifies the influential species and kinetic parameters that specifically modulate the pERK and pAkt responses, which represent potential targets for angiogenesis-based therapies.

**Conclusions:**

Overall, the model predicts the combination effects of FGF and VEGF stimulation on ERK and Akt quantitatively and provides a framework to mechanistically explain experimental results and guide experimental design. Thus, this model can be utilized to study the effects of pro- and anti-angiogenic therapies that particularly target ERK and/or Akt activation upon stimulation with FGF and VEGF.

**Video Abstract**

## Background

Angiogenesis is the formation of new blood capillaries from pre-existing blood vessels. The essential role of blood vessels in delivering nutrients to tissues makes angiogenesis important in many different settings, including both physiological and pathological conditions. Physiologically, angiogenesis is involved in the growth of normal blood vessels during development such as placental vascularization during pregnancy [1, 2] and the wound healing process [3, 4]. Pathological angiogenesis is crucial in many diseases, including cancer [5]. Thus, targeting angiogenesis is a prominent strategy in many contexts, for example, in both tissue engineering and cancer treatment. In the context of tissue engineering, researchers have sought to create artificial tissues to substitute damaged tissues in response to a great shortage of donors for transplant surgery. Implementing strategies that promote the formation of adequate vasculature is critical for the long-term viability of engineered tissue constructs. Therefore, stimulating new blood vessel formation is an important strategy for tissue engineering [6]. On the other hand, inhibiting angiogenesis is a strategy for cancer treatment, as the formation of new blood vessels is important for cancer growth and metastasis. Therefore, understanding the angiogenesis process is very beneficial to current strategies that target vessel formation.

Many different pro-angiogenic growth factors, such as fibroblast growth factor (FGF), vascular endothelial growth factor (VEGF), and platelet-derived growth factor (PDGF), mediate angiogenesis [7, 8]. These factors promote different cellular processes involving endothelial cells leading to new blood vessel formation, including proliferation, migration, survival, and vessel maturation [9, 10]. Strategies to promote or inhibit angiogenesis focus on modulating the effects of the factors that promote these cellular-level processes.

Unfortunately, not all approaches to promote or inhibit angiogenesis lead to successful outcomes. For example, clinical trials have shown no effective improvement in angiogenesis upon stimulation by FGF [11] or VEGF [12]. Also, bevacizumab, an anti-angiogenic agent designed to sequester VEGF extracellularly, inhibiting VEGF-mediated signaling by preventing VEGF from binding to its receptor [13, 14], has limited effects in certain cancer types, and it is no longer approved for the treatment of metastatic breast cancer due to disappointing results in patients [15]. Thus, there is a need to better understand the molecular interactions and signaling required for new blood vessel formation, in order to establish more effective therapeutic strategies.

Given the complex set of biochemical reactions comprising angiogenesis signaling networks, it is essential to apply computational modeling to better understand the dynamics of these networks. Computational modeling serves as a powerful tool to investigate molecular responses mechanistically and to guide experimental design. Indeed, many models have been developed to explore the angiogenic response mediated by growth factors. Models focused on the extracellular-level interactions [16–19] enhance our understanding of the distribution of angiogenic factors, which affects downstream angiogenic signaling. These models can be used to study strategies that regulate the distribution of angiogenic factors in tumor tissue. For example, Li and Finley constructed a compartmental whole-body model to study the effect of anti-angiogenic therapies targeting VEGF and TSP1 signaling in a simulated cancer patient cohort [18]. Also, models that study intracellular signaling [20, 21] can help identify potential targets and explore their efficacy for pro- or anti-angiogenic therapies.

Modulating angiogenesis signaling networks can involve targeting multiple angiogenic factors. There are few models that simulate the effects of more than one factor on intracellular signaling reactions at a detailed level. However, this insight is needed to better understand the effect of the angiogenic factors, mechanistically study experimental data, and guide new experiments. Moreover, in the case of inhibiting angiogenesis, tumors often evade the effects of drugs that target a single factor by making use of alternate compensatory pathways to activate signaling species needed for proliferation and migration. For instance, FGFR activation may play a role in the resistance mechanism of anti-angiogenic drugs, especially anti-VEGF treatment [22, 23]. Additionally, experiments show high levels of FGFR1 in tumors that continue to progress, even during anti-VEGF therapy [24]. FGF and VEGF have been shown to be particularly important in the early stages of angiogenesis, and we are interested in signaling crosstalk between these factors required to initiate vessel growth. Thus, we aim to quantitatively investigate the combination effects of FGF and VEGF on activating signaling in endothelial cells and identify potential intracellular targets by building a molecular-detailed computational model that incorporates the crosstalk between these pathways.

Specifically, in our model, FGF and VEGF bind to their receptors and initiate signaling through the mitogen-activated protein kinase (MAPK) and phosphatidylinositol 3-kinase/protein kinase B (PI3K/Akt) pathways to phosphorylate ERK and Akt, respectively. Previously, we studied the response of phosphorylated ERK (pERK) upon stimulation by FGF and VEGF [25], using mathematical modeling to gain insight into proliferation signaling, one aspect of the early stage of angiogenesis. However, angiogenesis involves not only proliferation, but also survival and migration of the endothelial cells. To get a more comprehensive understanding of this process, we expand the model to now incorporate the PI3K/Akt pathway, which has been shown to play an important role in cell survival [26–30] and migration [30–32]. Thus, in this study we examine the responses of pERK and phosphorylated Akt (pAkt) following mono- and co-stimulation by FGF and VEGF using mathematical modeling. The model predicts that the maximum pERK level is more responsive to changing the ligand concentration compared to the maximum pAkt level for certain concentration ranges. Also, co-stimulation with FGF and VEGF indicates a greater effect in increasing the maximum pERK compared to the summation of individual effects, which is not seen for maximum pAkt levels. Using this model, we also identified the influential species and kinetic parameters that specifically regulate the pERK and/or pAkt responses, indicating potential targets for pro- or anti-angiogenic therapies. The model predictions provide mechanistic insight into FGF and VEGF interactions in angiogenesis signaling. More broadly, this model provides a framework to study the efficacy of angiogenesis-based therapies.

## Methods

### Model construction

We constructed a molecular-detailed model that describes the intracellular network of FGF- and VEGF-induced ERK and Akt phosphorylation in endothelial cells. The model significantly expands on previous modeling work studying ERK [25] and Akt [21]. In our model, FGF binding to FGF receptor 1 (FGFR1) and heparan sulfate glycosaminoglycan (HSGAG) activates FRS2 and then initiates PI3K/Akt pathway, and VEGF binding to its receptor, VEGFR2, phosphorylates VEGFR2 and activates PI3K directly. In addition, activated FRS2 and Raf trigger MAPK pathway upon stimulation by FGF and VEGF, respectively.

**Fig. 1:**
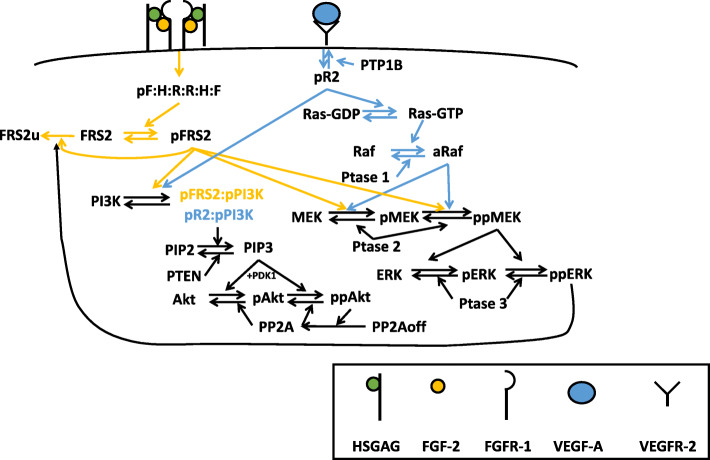
Schematic of FGF and VEGF signaling network. Signaling is induced by growth factors binding to their receptors, culminating with phosphorylation of ERK and Akt, through the MAPK and PI3K/Akt cascades, respectively

The molecular interactions involved in the network are illustrated in Fig. 1. This network is implemented as an ordinary differential equation (ODE) model using MATLAB. The main model includes 97 reactions, 99 species, and 100 parameters (see Supplemental file S3). The reactions, initial conditions and parameter values are listed in Tables S1-S3. All reactions are assumed to follow the law of mass action. Receptor internalization, recycling, and degradation are considered in the model, as these processes occur on a relatively fast timescale. However, because the simulated time is within 2 hours, we do not consider the degradation of the ligands or signaling species.

We note that the concentrations of extracellular ligands and intracellular species are considered with different relative volumes. Specifically, the concentrations of extracellular ligands are expressed relative to the volume of the cell culture media, while the concentrations of intracellular species are usually considered in a volume of a cell. In this study we focused on endothelial cells, which has been reported to have a mean cell volume of 1009 ± 180 *μ* m^3^ (1.01 ± 0.18 pL) [33]. Therefore, we used 1 pL cell volume to convert concentrations of intracellular species from molecules/cell to nM. This same conversion factor has been used in other computational work to study endothelial cell signaling responses [21, ﻿34﻿]. Details regarding interconversion between these units are provided in File S1.

### Sensitivity analysis

Before fitting the model to experimental data, we first performed a sensitivity analysis to identify the parameters and initial concentrations that significantly influence the model outputs for model training, using the extended Fourier Amplitude Sensitivity Test (eFAST) [35] method. Since the parameters and initial values for ERK activation were fit to experimental data in our previous model [25], we used the best fit values and held them constant during the sensitivity analysis. All remaining model parameters and initial values were varied simultaneously within two orders of magnitude above and below the baseline values, where the baseline values were taken from published literature [21]. In this way, the effects of multiple model inputs (kinetic parameters or initial conditions) on the pERK and pAkt concentrations were computed (the total sensitivity indices, “*Sti*”). The *Sti* values can range from 0 to 1, where a higher *Sti* index indicates the input is more influential to the output. Based on the experimental data that were used for model training, we calculated the *Sti* values using eFAST for all the same concentrations and time points as what was used in the experiments. The highest *Sti* value (*Sti*_*max*_) across all of the concentrations and time points was selected to represent the sensitivity index for each variable.

We also performed eFAST for the trained and validated model to identify potential targets for pro- and anti-angiogenic strategies. All parameters and initial concentrations were varied simultaneously within two orders of magnitude above and below the baseline values. In this case, the baseline values for the fitted variables were the median values estimated from model fitting. We calculated the *Sti* values to quantify how all the variables affected pERK and pAkt. Based on the behaviors of maximum pERK and pAkt that reach a plateau as the FGF and/or VEGF concentration increases, we selected five representative concentrations to capture the low (0.01 nM and 0.05 nM), intermediate (0.1 nM), and high (0.5 nM and 1 nM) levels of responses. We calculated the *Sti* values using eFAST for these five concentrations of FGF and VEGF stimulation at 15 time points ranging from zero to 120 min. Again, the *Sti*_*max*_ across all the concentrations and time points were compared for all the variables.

### Data extraction

Data from published experimental studies [36–41] were used for parameter fitting and model validation. The Western blot images were analyzed using ImageJ. Experimental data from plots was extracted using the *grabit* function in MATLAB.

### Model fitting and validation

Nine influential variables with *Sti*_*max*_ values greater than 0.8 were identified by performing eFAST (Table S4). The value of 0.8 was chosen as the cutoff to balance the fitting results and the computational expense. However, this included two correlated parameters, the kinetic rates *k_pFRS2PI3K* and kd_pFRS2PI3K, which are the forward and reverse rates, respectively, of the reaction pFRS2 + PI3K ↔ pFRS2:pPI3K. Thus, we held *k_pFRS2PI3K* constant and fitted the rest of the influential variables (Table S4).

Therefore, a total of eight variables (four initial conditions and four kinetic parameters) were estimated by fitting the model to experimental data using Particle Swarm Optimization (PSO) [42]. PSO starts with a population of initial particles (parameter sets). As the particles move around (i.e., as the algorithm explores the parameter space), an objective function is evaluated at each particle location. Particles communicate with one another to determine which has the lowest objective function value. The objective function for each parameter set was used to identify optimal parameter values. Specifically, we used PSO to minimize the weighted sum of squared residuals (WSSR):

$$ \mathrm{WSSR}\left(\uptheta \right)=\mathit{\min}\sum \limits_{i=1}^n{\left(\frac{V_{pred,i}\left(\uptheta \right)-{V}_{\mathit{\exp},i}}{V_{\mathit{\exp},i}}\right)}^2 $$

where V_*exp,i*_ is the *i*th experimental measurement, V_*pred,i*_ is the *i*th predicted value at the corresponding time point, and *n* is the total number of experimental data points. The minimization is subject to *θ*, the set of upper and lower bounds on each of the fitted parameters. The bounds were set to be two orders of magnitude above and below the baseline parameter values, which were taken from literature.

**Fig. 2:**
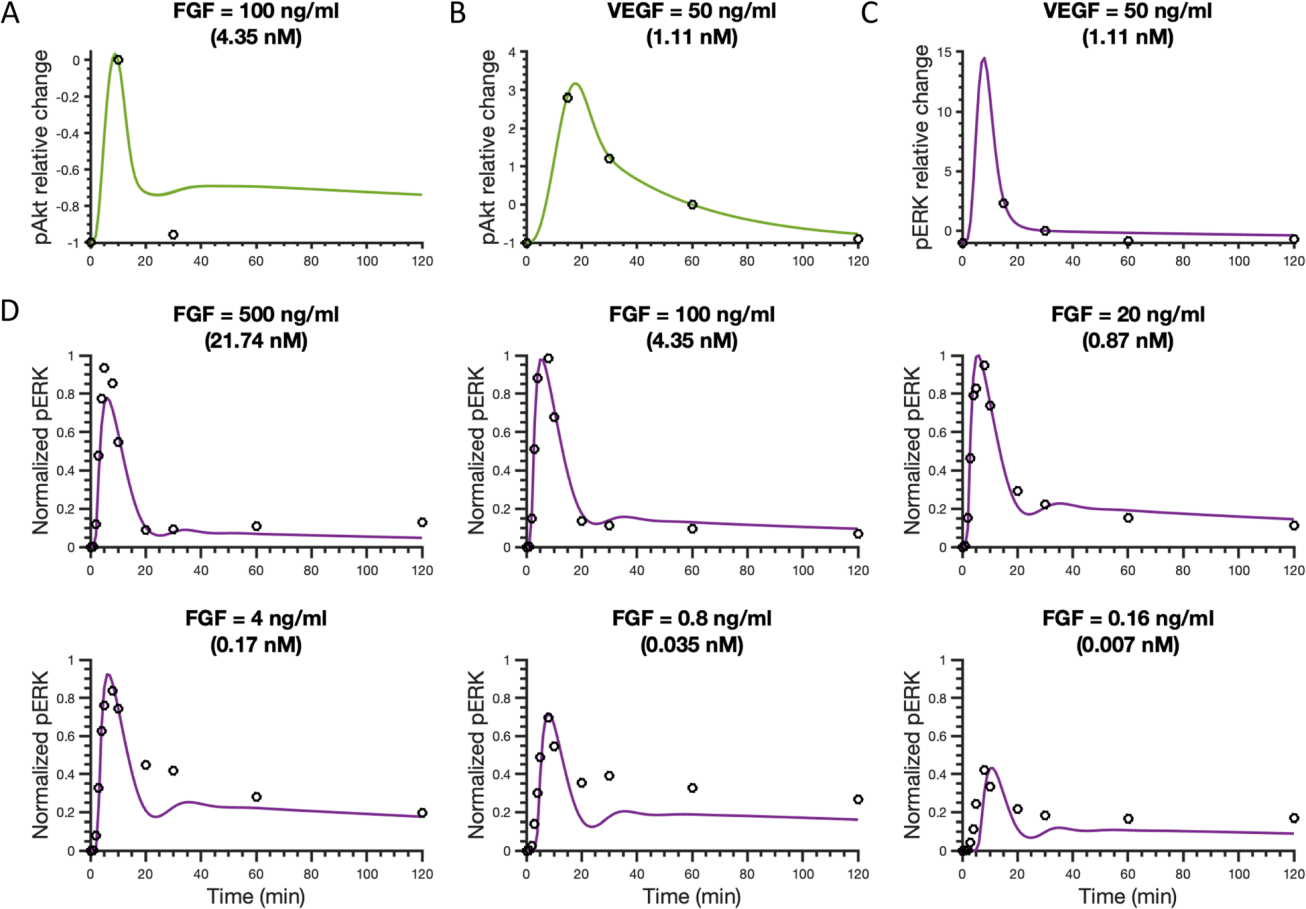
Model comparison to training data for FGF or VEGF stimulation. **a** Relative change of pAkt for 100 ng/ml (4.35 nM) FGF stimulation compared with the reference time point (10 min). **b** Relative change of Akt phosphorylation upon stimulation with 50 ng/ml (1.11 nM) VEGF compared with reference time point (60 min). **c** Relative change of ERK phosphorylation following stimulation with 50 ng/ml (1.11 nM) VEGF compared to the pERK level at a reference time point (30 min). **d** Normalized pERK dynamics in response to FGF concentrations ranging from 0.16 to 500 ng/ml (0.007–21.74 nM), where pERK level was normalized by the maximum pERK stimulated by FGF across all six concentrations in 2 hours. Circles in Panels A-C are experimental data from HUVECs, and circles in Panel D are experimental data from the NCI-H1730 cell line. Curves are the mean model predictions of the 15 best fits. Shaded regions show standard deviation of the fits

The model was fitted using four datasets as shown in Fig. 2, represented by circles. We note that the datasets shown in Fig. 2c and d were also used for fitting pERK levels in our previous work [25]. Here, we wanted to ensure that the model can still match this data, even upon expanding the model to include the PI3K/Akt pathway.

Model simulations were compared to experimental measurements. Specifically, the relative change of the responses was calculated as following:

$$ \mathrm{relative}\ \mathrm{change}\left(\mathrm{t}\right)=\frac{response\left(\mathrm{t}\right)- response\left({t}_{ref}\right)}{response\left({t}_{ref}\right)} $$

where *response*(t) is the level of pERK, pAkt, or phosphorylated VEGFR2 (pR2) at time *t*, and *response*(*t*_*ref*_) is the response (pERK, pAkt, or pR2) at a reference time point *t*_*ref*._ Here, the pERK and pAkt in the model simulation include all free and bound forms of singly- and doubly- phosphorylated ERK and Akt, respectively.

We note that the FGF-induced pERK response reported by Kanodia et al. was measured using the non-small cell lung cancer cell line NCI-H1730, while the rest of experimental measurements mentioned above were obtained using human umbilical vein endothelial cells (HUVECs). As in previous work [25], we assumed the FGFR1 signaling kinetics for NCI-H1730 are the same as in HUVECs, as FGFR1 and HSGAG levels are fairly consistent for various cell types [36, 43–45].

We first fitted the model 40 times to experimental data. However, we noticed that for the parameter sets with the lowest error, the fitted values for the *k_pFRS2fPIP3* parameter were all at the upper bound. To exclude the possibility of arbitrary bounds limiting the parameter searching space, we took this upper bound (20 s^− 1^) as the baseline value and expanded the bounds for this parameter to be 0.2–2000 s^− 1^. The targeted variables were estimated another 40 times with the new bounds. With this second round of fitting, none of the parameters were estimated to be at one of the bounds (Table S5).

After model training, we validated the model with three datasets not used in the fitting. We first predicted the VEGF-induced pR2 relative change upon stimulation with 80 ng/ml (1.78 nM) VEGF [39]. We also simulated the change of pAkt upon stimulation with 10 ng/ml (0.43 nM) FGF- [40] or 20 ng/ml (0.44 nM) VEGF-induced [41], respectively.

For all three datasets, we simulated the experimental conditions without any additional model fitting and compared to the experimental measurements. A total of 15 parameter sets with the smallest errors were taken to be the “best” sets based on the model fitting and validation (Figure S2 and Table S5) and were used for all model simulations.

### Monte Carlo simulations

To study the robustness of the system, we ran the fitted model 1000 times by generating 1000 values for all parameters and non-zero initial concentrations, sampling from a normal distribution. For initial concentrations and parameters that were estimated by fitting to the experimental data, the mean values (μ) were the median of the fitted values, and we used the standard deviation (σ) calculated from the fitted parameter sets. For all other model variable values, we set μ to be the baseline values and calculated σ to capture 99.7% of the possible values given the range of μ ± 50%μ (i.e., μ ± 3σ). It is worth noting that with this sampling, it is possible to get negative values, though this is unlikely to occur. However, if any negative values were selected, we resampled until all the sampled variables are positive.

### Signaling responses

We investigated the ERK and Akt phosphorylation responses upon stimulation by FGF or VEGF alone or in combination.

- *Maximum pERK and pAkt*. In our model simulations, for simplification, representative values were used as indicators for the magnitude of pERK or pAkt responses, specifically the maximum values. We calculated the maximum ERK and Akt phosphorylation levels induced by the stimulation by FGF, VEGF, or their combination.
- *Ratio, R*. To compare the combination effects to the effects of FGF and VEGF individually, we introduce the ratio below:

$$ R(response)=\frac{\mathit{\max}\ \mathrm{response}\left(\mathrm{FGF}\ \mathrm{and}\ \mathrm{VEGF}\right)}{\mathit{\max}\ \mathrm{response}\left(\mathrm{FGF}\right)+\mathit{\max}\ \mathrm{response}\left(\mathrm{VEGF}\right)} $$

When *R* is greater than one, it indicates that the combination effect in inducing the maximum response is greater than the summation of individual effects; when *R* is equal to one, it implies that the combination effect is additive; when *R* is less than one, it means that the combination of FGF and VEGF effect does not surpass the summation of individual effects and implies a competitive effect.

c. *Fold change, F*. To explore the efficiency of varying the identified influential variables, we define *F* as the predicted maximum pERK and pAkt levels when the parameters in Table 2 were varied by 0.1- or 10-fold individually compared to the baseline values. When *F* is greater than one, varying the parameter enhances the response; when *F* is equal to one, varying the parameter has no effect on the response; when *F* is less than one, it indicates that varying the parameter inhibits the response.

## Results

### The fitted and validated molecular-detailed mathematical model captures the major characteristics of FGF- and VEGF-induced ERK and Akt phosphorylation dynamics

We developed an intracellular signaling model of the crosstalk between two pro-angiogenic factors, FGF and VEGF. The signaling is initiated by FGF binding to FGFR1 and HSGAGs or VEGF binding to VEGF receptor 2 (VEGFR2), both promoting downstream signaling (Fig. 1). The model focuses on FGF- and VEGF-induced signaling through the MAPK and PI3K/Akt pathways, leading to activation of ERK and Akt, respectively. We consider that activated ERK and Akt include both the singly and doubly phosphorylated forms of each species (i.e., pERK and ppERK, as well as pAkt and ppAkt). For simplicity, we collectively refer to these species as phosphorylated ERK and Akt, pERK and pAkt, respectively. The model reactions, initial conditions, and parameter values are given in Supplementary Tables S1 to S3.

The parameters and initial concentrations involved in FGF- and VEGF-initiated MAPK signaling are taken from the “best” fit from our previous model [25]. As a starting point, the newly introduced parameters and initial concentrations involved in the PI3K/Akt pathway are acquired from literature [21]. The influential model parameters and initial conditions were estimated by fitting the model to experimental data, as described below.

For model training, we aimed to first identify the model variables (kinetic parameters and initial concentrations) that significantly influence the model outputs, phosphorylated ERK and Akt. To do so, we performed the eFAST sensitivity analysis [35] (see Methods for more details) and analyzed the total sensitivity indices (*Sti*) for the species’ concentrations and kinetic rates that are involved in the PI3K/Akt pathway. The highest *Sti* values (*Sti*_*max*_) across all of the concentrations and time points for the 32 newly introduced variables were compared, and nine of them (Table S4) were identified as influential to pERK and/or pAkt induced upon stimulation by FGF and VEGF. Of these, eight were not correlated (denoted by red text in Table S4), and we estimated their values by fitting the model to experimental measurements [36–38] using PSO [42] (see Methods for more details).

The fitted model shows a good agreement with experimental results (Fig. 2). It quantitatively captures the FGF- and VEGF-induced pAkt dynamics from experimental observations [37, 38] (Fig. 2a-b). In addition, this expanded model retains the ability to reproduce the measured pERK levels promoted by VEGF or FGF stimulation (Fig. 2c-d), including the biphasic pERK dose response following stimulation with FGF (Figure S1) reported by Kanodia et al. [36], which our previous model also reproduced. The weighted errors for 15 best fits are all approximately 20.7 (Table S5). Also, the estimated values of the fitted variables show good consistency (Figure S2).

**Fig. 3:**
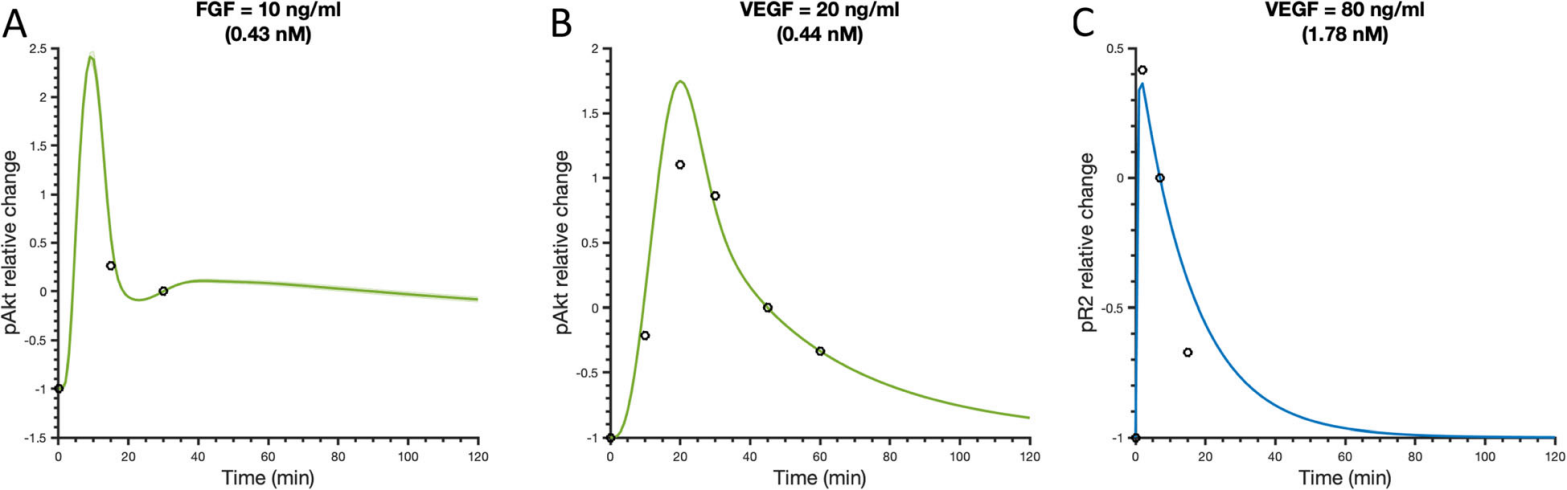
Model comparison to validation data. **a** Relative change of pAkt upon stimulation with 10 ng/ml (0.43 nM) FGF compared with reference time point (30 min). **b** Relative change of Akt phosphorylation upon stimulation with 20 ng/ml (0.44 nM) VEGF compared with reference time point (45 min). **c** Relative change of VEGFR2 phosphorylation upon stimulation with 80 ng/ml (1.78 nM) VEGF using the reference time point of 7 min. Circles are experimental data from HUVECs. Curves are the mean model predictions of the 15 best fits from the model training. Shaded regions show standard deviation of the fits

In addition to matching data used for fitting, the model is consistent with independent experimental observations. To validate the model, the predictions were compared to three independent sets of experimental data (Fig. 3). The model-predicted pAkt dynamics with 10 ng/ml (0.43 nM) FGF- or 20 ng/ml (0.44 nM) VEGF-induced pAkt agree with additional experimental data from Pisanti et al., 2011 [40] and Schneeweis et al., 2010 [41], respectively (Fig. 3a-b). In addition, model predictions match the levels of VEGFR2 phosphorylation following stimulation with 80 ng/ml (1.78 nM) VEGF extracted from a separate set of data from Chabot et al., 2009 [39] (Fig. 3c).

We performed Monte Carlo simulations (see Methods for more details) to investigate the predicted pERK and pAkt levels given variability in the initial conditions and parameters. The model predictions with parameters values randomly varied within the range of the estimated values can still capture pERK, pAkt, and pR2 dynamics stimulated by FGF and VEGF (Figures S3 and S4). These Monte Carlo simulations suggest that the overall dynamics of the model outputs, pERK and pAkt, are relatively robust to variability or uncertainty in initial species’ concentrations and parameters in the signaling network.

### FGF produces greater maximum pAkt and pERK than VEGF at equimolar concentrations

**Fig. 4:**
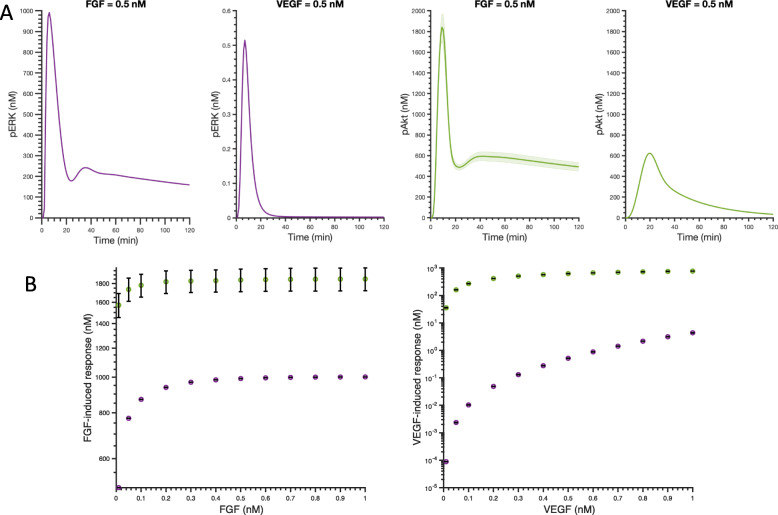
Predicted pERK and pAkt responses stimulated by single agents. **a** Predicted time courses of pERK and pAkt stimulated by 0.5 nM FGF and 0.5 nM VEGF. Curves are the mean predictions for the 15 best fits from the model training. Shaded regions show standard deviation of the fits. **b** Maximum pERK (Purple) and pAkt (Green) in response to FGF (left) and VEGF (right) for concentrations varying from 0.01 nM to 1 nM. Bars are mean ± standard deviation of model predictions. Note that the y-axes are not on the same scale

We first explored the individual effects of FGF and VEGF on pERK and pAkt using the trained and validated model. The dynamics of pERK and pAkt stimulated by 0.5 nM FGF and 0.5 nM VEGF are shown in Fig. 4a. The model predicts transient activation of ERK and Akt following stimulation by FGF or VEGF. The species’ concentrations are predicted to peak within 30 min and return to basal level after 60 min, as seen in experimental data used for model fitting and validation. These predicted time courses show that 0.5 nM FGF stimulation leads to higher maximum levels of pERK and pAkt, compared to 0.5 nM VEGF stimulation.

We also simulated the pERK and pAkt dynamics for a range of concentrations of FGF or VEGF. Here, we use the maximum pERK and pAkt levels within the two hours simulated by the model as indicators for pERK and pAkt responses. Maximum pAkt (green) and pERK (purple) levels increase with the increase of FGF or VEGF concentrations (Fig. 4b). The model predicts that the average levels of the maximum pAkt and pERK across the 15 best fits, given 0.5 nM FGF stimulation, are 1.8 × 10^3^ nM and 1.0 × 10^3^ nM, respectively. In comparison, 0.5 nM VEGF induces an average maximum pAkt and pERK of 6.3 × 10^2^ nM and 5.2 × 10^− 1^ nM, respectively. Thus, 0.5 nM FGF produces averaged maximum Akt and ERK phosphorylation levels that are 3-fold and nearly 2000-fold higher than that induced by 0.5 nM VEGF, respectively.

We can use the detailed model to explain these results. In our previous work [25], we described that the main reasons that FGF induces a greater maximum pERK response compared to VEGF are the relatively high level of FGFR density compared to VEGFR2 (33.2 versus 1.7 nM; 20,000 versus 1000 molecules/cell, respectively) and lower internalization and degradation rates for FGFR compared to the corresponding VEGFR2 parameters. These differences also make FGF-induced pAkt higher than VEGF-induced pAkt. Indeed, the model predicts that increasing VEGFR2 level by 10-fold can increase the 0.5 nM VEGF-induced maximum pAkt to approximately the same maximum pAkt level induced by 0.5 nM FGF (Figure S5A). In addition, decreasing VEGFR2 internalization and degradation rates to be the same level as the corresponding FGFR internalization and degradation rates leads to an increase in VEGF-induced maximum pAkt level (Figure S5B). This is because lower internalization and degradation rates lead to more signaling complexes available for signal transduction. Together, these receptor-related properties (density, internalization, and degradation) lead to stronger signaling induced by FGF.

### Akt activation shows a stronger response than ERK in terms of magnitude

**Fig. 5:**
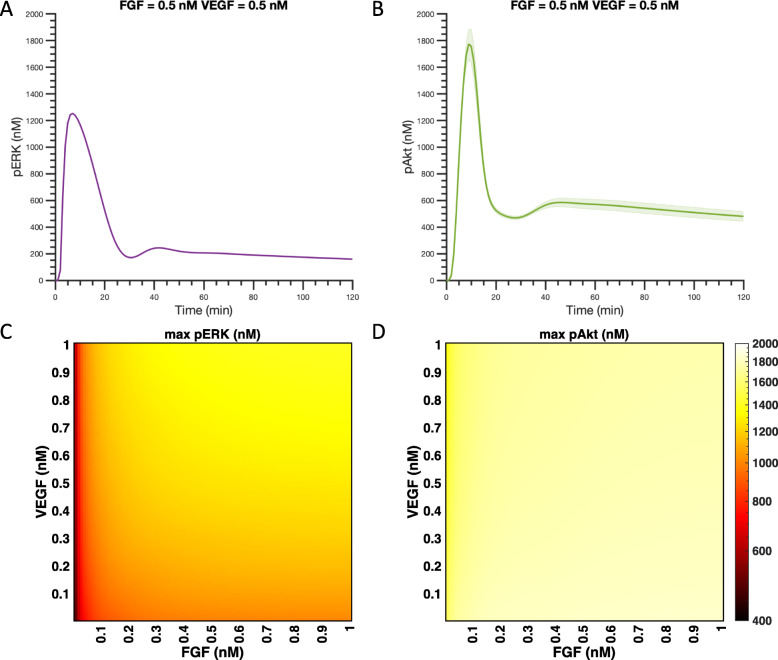
Predicted maximum pERK and pAkt responses with co-stimulation. Predicted time courses of pERK (**a**) and pAkt (**b**) stimulated by the combination of 0.5 nM FGF and 0.5 nM VEGF. Curves are the mean predictions for the 15 best fits from model training. Shaded regions show standard deviation of the fits. Maximum pERK (**c**) and pAkt (**d**) in response to co-stimulation by FGF and VEGF for concentrations varying from 0.01 nM to 1 nM

We also compared Akt and ERK activation responses by the mono- and co-stimulation of FGF and VEGF. The model predictions show that the maximum pAkt level is higher than the maximum pERK level in response to same ligand concentration, whether considering FGF or VEGF stimulation (Figs. 4b and 5). As shown in Fig. 4b, when FGF concentration is varied from 0.01 to 1 nM, the averaged maximum pAkt and pERK range from 1.6 × 10^3^ nM to 1.8 × 10^3^ nM and 5.0 × 10^2^ nM to 1.0 × 10^3^ nM, respectively. Similarly, for the same concentration range of 0.01–1 nM, VEGF induces an averaged maximum pAkt of 3.5 × 10^1^ nM to 7.6 × 10^2^ nM pAkt and maximum pERK of 8.8 × 10^− 5^ nM to 4.3 × 10^0^ nM (Fig. 4b). Thus, for FGF mono-stimulation, the maximum pAkt level induced by low FGF concentration (0.01 nM) is even higher than the maximum pERK level activated by a high concentration of FGF (1 nM). The same holds true for VEGF mono-stimulation – a low concentration of VEGF (0.01 nM) induces a higher maximum pAkt than the maximum pERK stimulated by high VEGF concentration (1 nM).

We then studied the effects of co-stimulation of FGF and VEGF in inducing maximum pERK and pAkt. The dynamics of pERK and pAkt stimulated by 0.5 nM FGF and 0.5 nM VEGF in combination are shown in Fig. 5a and b, respectively. Similar to mono-stimulation, the model also predicts transient activation of ERK and Akt by the co-stimulation of FGF and VEGF. The dynamics of pERK and pAkt are predicted to reach their maximum level within 30 min and return to basal level after 60 min. We also predicted the averaged maximum pERK and pAkt induced by co-stimulation of FGF and VEGF in a range of 0.01–1 nM across the 15 best fits (Fig. 5c-d). Maximum pAkt shows a greater response at all combinations of FGF and VEGF stimulation compared to the maximum pERK induced by the same combinations (Fig. 5c-d).

Studying the model mechanistically, we found that the main reason that Akt showed a higher level of activation compared to ERK is because the initial concentration of PP2A, the phosphatase that acts on pAkt (2.5 nM), is much lower than the initial concentrations of the phosphatases that act on pMEK and pERK, Ptase2 and Ptase3 (3.7 × 10^2^ and 1.7 × 10^3^ nM, respectively). In the model, PP2A can be produced by PP2Aoff (see reactions R87 and R88 in Table S1); however, the initial amount of PP2Aoff (1.1 × 10^2^ nM) is not high enough to make the PP2A level comparable to that of Ptase2 and Ptase3.

To confirm the effect of phosphatases, we decreased Ptase2 and Ptase3 levels to be 2.5 × 10^0^ nM, which is the same level as PP2A. The model predicts that when Ptase2 and Ptase3 levels are decreased, stimulation by FGF, VEGF, or their combination in the range of 0.01–1 nM induced 5.6 × 10^2^–8.3 × 10^2^, 3.4 × 10^1^–7.6 × 10^2^, and 5.6 × 10^2^–9.6 × 10^2^ nM for maximum pAkt, respectively. In comparison, the maximum pERK is predicted to be 3.5 × 10^3^, 9.5 × 10^1^ – 3.5 × 10^3^, and 3.5 × 10^3^ nM, respectively, for these three cases, as pERK saturated to reach its maximum level such that all of the ERK initially present (3.5 × 10^3^ nM) was phosphorylated. Thus, the predicted maximum pERK level surpassed maximum pAkt when Ptase2 and Ptase3 levels decreased, confirming that the relatively high levels of Ptase 2 and Ptase3 limit the ERK phosphorylation compared with Akt.

### ERK activation is more responsive to changing the ligand concentration compared to Akt

We compared pERK and pAkt behaviors in terms of their responsiveness (i.e., sensitivity) to changes in FGF and VEGF concentrations. The fold change of pAkt for VEGF stimulation of 0.01 nM compared to 1 nM for the baseline model is predicted to be 21.7, while the fold change of pERK is 4.9 × 10^4^. The fold changes for pAkt and pERK for FGF stimulation at 0.01 nM compared to 0.4 nM are 1.2 and 2.0. In addition, there appears to be an optimal ligand concentration required to attain the maximum response for FGF-induced pERK and pAkt, as their dose-response curves plateau at approximately 0.4 nM and 0.1 nM, respectively, for stimulation with a ligand concentration in the range of 0.01 nM – 1 nM FGF (Fig. 4b). The maximum level of pAkt also plateaus at 0.4 nM VEGF. Before the response saturates, maximum pERK shows a steeper increase than maximum pAkt for increasing levels of either FGF or VEGF (Fig. 4). In addition, maximum pERK following stimulation by VEGF continues to increase for the concentration range simulated here (Fig. 4b). Altogether, these results indicate that pERK is more responsive to changing the ligand concentrations, as compared to pAkt in the range of 0.01 nM to the saturation concentration.

We next studied the effects of FGF and VEGF in combination on pERK and pAkt. The maximum pERK attained upon co-stimulation shows an increase with increasing FGF and VEGF (Fig. 5c). In contrast, maximum pAkt induced by co-stimulation with FGF and VEGF is approximately the same for all of the combinations examined (Fig. 5d). Thus, the model suggests that with FGF and VEGF co-stimulation, phosphorylation of ERK is more dose-dependent compared to Akt activation, similar to modeling predictions for mono-stimulation.

Given its mechanistic detail, we can use the model to explain the reason for the greater sensitivity of maximum pERK compared to pAkt. We found that the reason Akt activation is less responsive to varying ligand concentrations is due to the interaction between pAkt and the phosphatase PP2A, both in terms of the binding rates and negative feedback. Regarding the binding rates between activated (phosphorylated) species and their phosphatases, we compare doubly phosphorylated Akt and doubly phosphorylated MEK, as these species are each in the first layer of phosphorylation reactions that lead to formation of doubly phosphorylated MEK and Akt following FGF or VEGF binding to their corresponding receptors. This layered structure of the phosphorylation reactions enables nonlinear signaling amplification. The association rate of ppAkt and PP2A (*k_aPP2A* = 1.0 × 10^− 3^ 1/nM/s) is two orders of magnitude lower than the association rate of ppMEK and the phosphatase Ptase2 (*k_dpMEK_pp* = 1.4 × 10^− 1^ 1/nM/s). This relatively low association rate of ppAkt and PP2A leads to accumulation of both pAkt and ppAkt, since the phosphorylated species bind slowly to the phosphatase. The accumulation of the phosphorylated Akt due to this lower association rate more strongly influences the levels of phosphorylated Akt compared to the effect of increasing the concentrations of the ligands. Therefore, the total pAkt is relatively stable in response to stimulation. In comparison, since the association rate of phosphorylated MEK and Ptase2 is faster, less phosphorylated MEK accumulates, and the system remains responsive to increases in the ligand concentrations.

Another contributor to the differences in activation of MAPK versus the PI3K/Akt pathway is negative feedback induced by phosphorylated Akt. Production of PP2A, the phosphatase that acts on pAkt and ppAkt, is promoted by ppAkt itself [21, 46] (see reactions R87 and R88 in Table S1). This feedback highly regulates pAkt and ppAkt levels such that when more ppAkt is produced, the level of PP2A also increases. The presence of this negative feedback loop makes phosphorylated Akt levels less responsive to increased ligand concentration. In contrast, the amounts of the phosphatases Ptase2 (which dephosphorylates MEK) and Ptase3 (which dephosphorylates ERK) do not depend on upstream species. Thus, varying the amount of FGF or VEGF does not affect the phosphatases’ concentrations.

We confirmed the influence of ppAkt-PP2A binding and PP2A negative feedback by systematically altering the network for Akt activation to be more like the network for ERK activation (Table 1). We first increased the association rate of doubly phosphorylated Akt and PP2A to be the same as the rate for doubly phosphorylated MEK and Ptase2 binding. For this case, the fold change of ppAkt comparing two levels of VEGF stimulation (1 nM versus 0.01 nM) is 6.0 × 10^3^. When the network is modified such that PP2A is not activated by doubly phosphorylated Akt, the fold change is predicted to be 3.4 × 10^3^. If the ppAkt-PP2A association rate is increased and negative feedback is removed, the fold change in ppAkt is 1.0 × 10^5^. Thus, in all three of these cases where the network is modified, the fold change for ppAkt is much higher than the baseline case. The fold change of ppAkt in response to two levels of FGF stimulation (0.4 nM versus 0.01 nM) is also predicted to be higher than the baseline case when the association rate of doubly phosphorylated Akt and PP2A is increased (Table 1). However, when the negative feedback is removed, Akt is used up, even by the stimulation of 0.01 nM FGF. Thus, the fold change of ppAkt in response to FGF stimulation is even lower than the baseline model because of the shortage of Akt. These simulations confirm that the unresponsiveness of pAkt to changes in ligand concentration is due to the particular properties of the Akt activation pathway. Overall, the model predictions and analysis provide quantitative insight that helps to better understand how the signaling response can be modulated to achieve a desired effect.

**Table 1:** Fold change for pAkt and pMEK in response to varying ligand concentration

	0.01 nM VEGF (nM)	1 nM VEGF ^a^ (nM)	Fold change (comparing the two ligand concentrations)	0.01 nM FGF (nM)	0.4 nM FGF ^b^ (nM)	Fold change (comparing the two ligand concentrations)
Doubly phosphorylated Akt						
Baseline model	4.88 × 10^−1^	1.91 × 10^2^	3.92 × 10^2^	7.75 × 10^2^	1.08 × 10^3^	1.39
Increase association rate of doubly phosphorylated Akt and PP2A	3.94 × 10^−3^	2.38 × 10^1^	6.03 × 10^3^	3.13 × 10^2^	5.45 × 10^2^	1.74
Remove PP2A negative feedback mediated by ppAkt	4.96 × 10^−1^	1.69 × 10^3^	3.41 × 10^3^	2.33 × 10^3^	2.35 × 10^3^	1.01
Increase association rate and remove negative feedback	3.94 × 10^−3^	3.96 × 10^2^	1.00 × 10^5^	2.31 × 10^3^	2.34 × 10^3^	1.01
Doubly phosphorylated MEK						
Baseline model	4.33 × 10^−6^	2.10 × 10^−1^	4.86 × 10^4^	2.48 × 10^1^	5.27 × 10^1^	2.12

^a^ 1 nM VEGF is the highest concentration considered in model simulations. The maximum pERK has not saturated at this ligand level

^b^ 0.4 nM FGF is used as the highest concentration since both maximum pERK and pAkt have saturated by this ligand level

### The co-stimulation by FGF and VEGF has a greater impact on phosphorylation of ERK compared to summation of the ligands’ individual effects

To explore how FGF and VEGF influence pERK and pAkt responses together, we compared the combination effects to the summation of individual effects in inducing maximum pERK and pAkt. The dynamics of pERK and pAkt stimulated by 0.5 nM FGF and 0.5 nM VEGF in combination (solid lines) and in summation (dashed lines) are shown in Figure S6. We observed greater pERK levels induced by the co-stimulation of 0.5 nM FGF and 0.5 nM VEGF compared to the summation of individual effects within before the activation gets attenuated. On the other hand, the summation of 0.5 nM FGF and 0.5 nM VEGF induced pAkt is greater than the co-stimulation at all simulated time. To more concisely represent the signaling response induced by the growth factors, we compared the maximum pERK and pAkt induced by FGF and VEGF co-stimulation to the summation of individual effects. We defined this ratio as *R*(response), where *response* stands for pERK or pAkt (see Methods for more details).

**Fig. 6:**
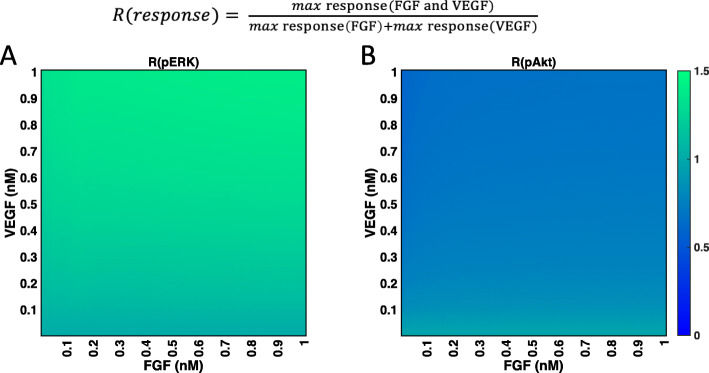
Comparison of mono- and co-stimulation. Ratios, *R*, comparing the combination effects to the summation of individual effects in response to FGF and VEGF for maximum pERK (**a**) and pAkt (**b**)

Figure 6a shows that *R*(pERK) is greater than one for combinations of FGF and VEGF ranging from 0.01 nM to 1 nM, specifically R(pERK) ranges from 1.01 to 1.44, and 53% of combinations that we simulated have R(pERK) greater than 1.25. This indicates that the combination effect of inducing maximum pERK is greater than the summation of individual effects, which is consistent with our previous work [25]. Our simulations show that 0.01 nM FGF induces a maximum of 5.0 × 10^2^ nM pERK, while 1 nM VEGF produces 4.3 nM pERK. Given this 100-fold difference, we believe a 25% increase in maximum pERK by the co-stimulation compared to FGF stimulation alone is significant. As explained in 3.2, the reason VEGF is not as potent as FGF in inducing maximum pERK could be due to the low VEGF receptor level and high trafficking parameters, compared to FGF receptors [25].

In contrast, *R*(pAkt) for combinations of FGF and VEGF concentrations ranging from 0.01 nM to 1 nM is less than one (Fig. 6b). This suggests that the combination of FGF and VEGF is not as effective in inducing maximum pAkt, as compared to the summation of the responses induced by each ligand individually.

We again applied the model to explain these predicted behaviors. We find that the values of *R*(pERK) are greater than one because the co-stimulation compensates for limitations observed when only one ligand is applied [25]. Because there is abundant Raf available in comparison to limited FRS2 level, VEGF co-stimulation helps to overcome the stoichiometric limitations of FRS2 for FGF mono-stimulation. Also, because pFRS2 phosphorylates MEK faster than aRaf, FGF co-stimulation provides a high level of pMEK. Therefore, FGF and VEGF co-stimulation exhibits a greater effect in phosphorylating ERK than the summation of individual effects.

The reason why the *R*(pAkt) values are less than one is due to features of the network. Specifically, phosphorylated Akt is more stable in response to the change of ligand concentration as a result of the low association rate for ppAkt and the phosphatase PP2A and the negative feedback of ppAkt promoting the production of its own phosphatase, PP2A. As explained in the previous section, with mono-stimulation, the pAkt level remains relatively constant, even as the ligands’ concentrations change. Additionally, the pAkt levels induced by combinations of FGF and VEGF are approximately the same as the pAkt levels induced by FGF alone (Figs. 4b and 5b). Thus, summing the individual effects to include the VEGF-induced maximum pAkt level means that the denominator in the ratio *R* is greater than the numerator, forcing *R* to be less than one.

We note that mono- and co-stimulation of FGF and VEGF affects not only the magnitude of pERK and pAkt levels, but also the time required to reach the maximum responses and the duration of the responses. The timescale of the pERK response was a major focus of our previous work [25]. Briefly, we found that the combination of FGF and VEGF exhibits a fast and sustained pERK response compared to mono-stimulation [25].

### The model identifies potential targets for influencing ERK and Akt activation and evaluates their efficacy

We applied the model to determine the parameters and initial concentrations that significantly influence pERK and pAkt levels, providing insight for researchers seeking to effectively modulate the MAPK and PI3K signaling pathways required for angiogenesis. We identified the influential model variables by evaluating the *Sti*_*max*_ calculated in the eFAST global sensitivity analysis (Figure S7 and Table 2) (see Methods for more details). As a rule of thumb, we consider variables to not be influential if their *Sti*_*max*_ values are lower than 0.5, and the variables that have *Sti*_*max*_ values greater than 0.7 are taken as influential. The influential variables (Table 2) could be potential targets for pro- or anti-angiogenic strategies. Interestingly, this analysis shows that species’ concentrations and kinetic parameters of the upstream signaling network are strong regulators for both pERK and pAkt levels and are shown to have high *Sti*_*max*_ values for both pERK and pAkt levels. This includes: initial concentrations of HSGAG, VEGFR2, and FGFR; kinetic parameters *kf5a*, *k_pR2*, and *kf0*, which are involved in ligand receptor binding reactions; as well as the parameter *k_1PI3K*, which is the association rate of pR2 and PI3K and plays an important role in the competition between the two pathways. Not surprisingly, species and kinetic parameters involved in intermediate or downstream signaling leading to ERK are influential and specific modulators of pERK. Similarly, we identify model variables that specifically influence Akt. For instance, *Sti*_*max*_ values of the MEK-Raf dissociation rate (*kd_aMEKRaf*), pMEK phosphorylation rate mediated by pFRS2 (*kf37*) and the ERK level are shown be greater than 0.7 for pERK but less than 0.1 for pAkt; while Akt and PI3K levels that are involved in PI3K/Akt pathway are only influential to pAkt.

**Table 2:** The total sensitivity index *Sti* values

	**Parameter name**	***Sti*values**^**a**^		**Description**
		**pERK**	**pAkt**	
**Only influential to pERK**	***kd_dpRaf***	0.87	0.00	Dissociation rate of Raf_a and Ptase1
	**[ERK]**	0.84	0.08	Initial concentration of ERK
	***kd_aMEKRaf***	0.84	0.00	Dissociation rate of MEK and Raf_a
	***kd_RasGDP***	0.83	0.00	Ras-GTP activation rate
	**[Ptase1]**	0.80	0.03	Initial concentration of Ptase1
	***kf37***	0.77	0.06	pMEK phosphorylation rate via pFRS2
	***ked_MEKRaf2***	0.77	0.00	pMEK phosphorylation rate via aRaf
	**[Ptase2]**	0.73	0.10	Initial concentration of Ptase2
	***k_aERKMEK***	0.73	0.05	ERK/pERK and ppMEK association rate
**Only influential to pAkt**	**[Akt]**	0.21	0.83	Initial concentration of Akt
	**[PI3K]**	0.44	0.80	Initial concentration of PI3K
	***k_fPIP3***	0.04	0.78	PIP3 activation rate via pR2:pPI3K:PIP2
	**[PP2Aoff]**	0.08	0.76	Initial concentration of active PP2A
	**[PIP2]**	0.27	0.73	Initial concentration of PIP2
	**[PTEN]**	0.22	0.72	Initial concentration of PTEN
	**[Ras-GDP]**	0.35	0.71	Initial concentration of Ras-GDP
**Influential to pERK and pAkt**	**[H]**	0.87	0.79	Initial concentration of HSGAG
	**[R2]**	0.86	0.83	Initial concentration of VEGFR2
	***kf5a***	0.84	0.71	FGFR and FGF:HSGAG association rate
	**[R]**	0.83	0.79	Initial concentration of FGFR
	**[FRS2]**	0.78	0.73	Initial concentration of FRS2
	[MEK]*	0.76	0.67	Initial concentration of MEK
	***k_pR2***	0.75	0.80	VEGFR2 phosphorylation rate
	kf0*	0.66	0.75	FGF and HSGAG association rate
	k_1PI3K*	0.65	0.81	Association rate of pR2 and PI3K

^a^ The variables that have *Sti* values greater than 0.7 are considered as influential, but not very influential if their *Sti* values are lower than 0.5. In the category of influential to pERK and pAkt, [MEK], *kf0*, and *k_1PI3K* are labeled with asterisks as they do not strictly meet these criteria; they have *Sti* > 0.7 for one output and 0.6–0.7 for the other

**Fig. 7:**
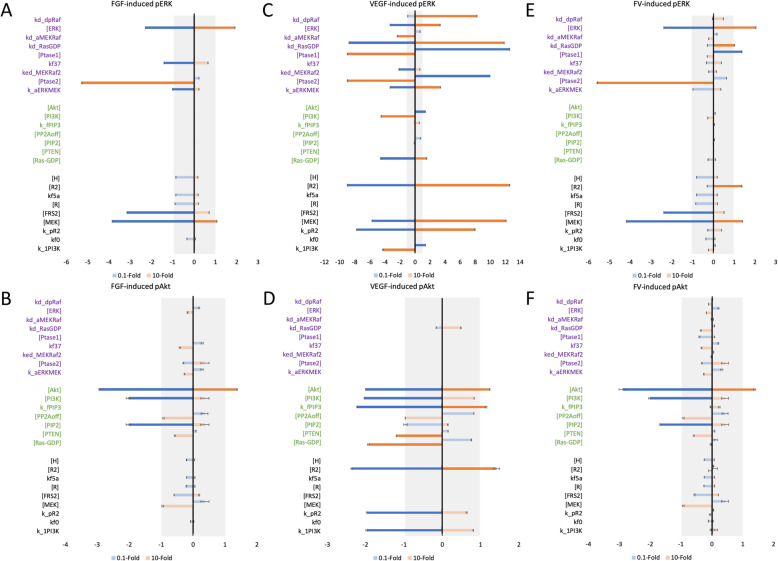
Predicted targets for modulating pERK and pAkt responses. Log_2_(*F*) for 0.5 nM FGF-induced pERK (**a**) and pAkt (**b**); 0.5 nM VEGF-induced pERK (**c**) and pAkt (**d**); and combination of 0.5 nM FGF- and 0.5 nM VEGF-induced pERK (**e**) and pAkt (**f**). x-axes are log_2_(*F*), y-axes are variables from Table 1. Bars are mean ± standard deviation of model predictions

The eFAST analysis tells which model variables the variances in the predicted pERK and pAkt can be attributed to. However, it is also important to determine how finitely changing those influential variables affects the output. That is, we aim to understand i) whether the influential variables promote or inhibit ERK and Akt activation and ii) how much the pERK and pAkt levels change when the influential variables are changed. Therefore, the ratios of the maximum pERK and pAkt levels compared to the baseline values were predicted when the parameters in Table 2 were varied by 0.1- and 10-fold (Fig. 7). This ratio is defined as the fold change, *F*, in the response (see Methods for more details). We consider the parameters that cause log_2_(*F*) to be greater than |1| (i.e., a two-fold change) as effective targets.

We calculated log_2_(*F*) for the variables in Table 2, identifying effective targets that modulate pERK and pAkt upon stimulation with 0.5 nM FGF or 0.5 nM VEGF, or with co-stimulation with 0.5 nM FGF and 0.5 nM VEGF. These predictions complement in vitro studies that focus on the responses induced by angiogenic agents. The model predicts that increasing ERK and MEK levels can strongly promote FGF- and VEGF-induced pERK (Fig. 7a and c), and increasing Akt promotes FGF- and VEGF-induced pAkt (Fig. 7b and d). Similarly, decreasing ERK, MEK and Akt can effectively inhibit pERK and pAkt. These predicted targets are intuitive, as they are directly related to the signaling species of interest.

Excitingly, the model predicts several other targets. For example, increasing the phosphatase Ptase2, which dephosphorylates pMEK and ppMEK, and decreasing FRS2, which is an upstream FGF-mediated signaling species, significantly inhibits FGF-induced pERK (Fig. 7a). Also, decreasing PIP2, the substrate for producing PIP3, which further phosphorylates Akt and pAkt, is another effective means of inhibiting FGF-induced Akt phosphorylation (Fig. 7b). In addition, our model predicts that the initial concentrations of VEGFR2 and Ras-GDP positively regulate VEGF-induced ERK phosphorylation (Fig. 7c), while increasing the VEGFR2 level and the PIP3 activation rate (*k_fPIP3*) are effective strategies to promote VEGF-induce pAkt (Fig. 7d). Also, increasing the concentrations of phosphatases Ptase1 and Ptase2, which deactivate Raf and MEK, respectively, can inhibit VEGF-induced pERK, as they are negative regulators of ERK phosphorylation (Fig. 7c). Increasing PTEN (the phosphatase for PIP3) and the Ras-GDP level can inhibit VEGF-induced pAkt as well (Fig. 7d). It is noteworthy that the model predicts that the VEGFR2 level and *k_pR2* positively regulate both VEGF-induced pERK and pAkt. Thus, VEGFR2 and *k_pR2* are targets for promoting or inhibiting the activation of both pathways mediated by VEGF.

Interestingly, the model predicts strategies to explore the effect on ERK and Akt activation individually. There are three potential targets that have opposing effects for the two pathways and can be utilized to enhance the signal transduction for one pathway and dampen the response of the other pathway. Also, these targets could play a role in the mechanism of resistance in which inhibiting one pathway enables greater activation of the other. Specifically, decreasing the PI3K level and the pVEGFR2 and PI3K association rate (*k_1PI3K*) can enhance VEGF-induced pERK, but reduces VEGF-induced pAkt. This opposing effect is because decreasing PI3K level and *k_1PI3K* can reduce the signal transduction for PI3K/Akt pathway, however decreasing PI3K level means there is relatively less PI3K (upstream species for PI3K/Akt pathway) competing against Ras-GDP (upstream species in the MAPK pathway) for pVEGFR2 induced by VEGF (Fig. 1). Also, decreasing pVEGFR2 and PI3K association rate (*k_1PI3K*) reduces the competition of PI3K/Akt pathway for pVEGFR2. Therefore, there is relatively more pVEGFR2 utilized for activating the MAPK pathway when the competition of PI3K/Akt pathway is reduced, and this further leads to an elevated pERK level induced by VEGF. In addition, increasing Ras-GDP level promotes VEGF-induced pERK but inhibits VEGF-induced pAkt. This is also caused by the competition between two pathways. Increased Ras-GDP level consumes more pVEGFR2, which limits PI3K activation by pVEGFR2 and further reduces pAkt level induced by VEGF.

Our model predicts other potential effective pro- and anti-angiogenic strategies (Fig. 7); however, it is of interest to investigate the effects of the crosstalk. Since FGF and VEGF are typically both present in physiological or pathological conditions, it is relevant to identify variables that affect activation of ERK and Akt upon co-stimulation with FGF and VEGF (Fig. 7e-f). The model shows that an increase in ERK, MEK, and VEGFR2 levels, as well as increasing the *kd_RasGDP* rate promotes ERK phosphorylation. Decreasing the Ptase1 level also enhances pERK. In addition, increasing the initial level of Akt is the most effective pro-angiogenic strategy to enhance Akt phosphorylation. On the other hand, increasing Ptase2, and decreasing the MEK, ERK, and FRS2 levels inhibit pERK. Lastly, decreasing the Akt, PI3K and PIP2 levels is effective anti-angiogenic strategies to inhibit pAkt.

Finally, we compared the effect of the identified effective potential targets under different treatments (FGF-, VEGF-, and FGF/VEGF-stimulation). Interestingly, we found that some potential targets predicted to have an effect in response to mono-stimulation had only limited effects in response to co-stimulation. For instance, increasing the initial level of Ptase1 was predicted to effectively inhibit VEGF-induced pERK (Fig. 7c); however, increasing Ptase1 leads to a log_2_(*F*) value of only − 0.3 (an 0.8-fold change) when we simulate FGF and VEGF co-stimulation. This implies that FGF-mediated signaling can diminish the inhibitory effect of increasing Ptase1 that occurs with VEGF-induced pERK, and further illustrates the effect of compensatory pathways in the overall results. Our simulations show that it is critical to study the network systematically to identify potential effective targets for specific conditions.

## Discussion

We developed an intracellular signaling model of the crosstalk between two pro-angiogenic factors, FGF and VEGF. The model focuses on pERK and pAkt responses as indicators for signaling promoted by the two pro-angiogenic factors. In this study, we built on our previous modeling work and incorporated PI3K/Akt pathway to get a more comprehensive understanding of the angiogenesis process, as the PI3K/Akt pathway is important in regulating cell survival [26–30] and migration [30–32].

Excitingly, the primary model predictions are supported by experimental students, providing confidence that the model can be used to examine novel aspects of FGF- and VEGF-mediated signaling. Our model predicts that the maximal levels of FGF- and VEGF-induced pERK and pAkt plateau as the ligand concentration increases. This prediction is supported by experimental studies showing an optimal concentration for FGF- and VEGF-induced human umbilical vein cells (HUVECs) tube formation on Matrigel for 24 h (0.1 ng/ml (0.004 nM) and 25 ng/ml (0.56 nM), respectively), also 0.1 ng/ml (0.004 nM) FGF exhibits approximately same level of increase in HUVECs proliferation and migration as 25 ng/ml (0.56 nM) VEGF stimulation [47]. In addition, the model predicts that the combination of FGF and VEGF stimulation induces ERK phosphorylation to a greater extent than the sum of the individual effects of FGF and VEGF. In contrast, the combination of FGF and VEGF does not promote enhanced Akt phosphorylation compared to the summation of the response stimulated by FGF and VEGF individually. These predictions are consistent with experimental observations.

Researchers have shown that endothelial sprouting is FGF and VEGF dose dependent [47, 48], and that the combination of FGF and VEGF induces greater total sprout length than summation of individual effects [48]. Goto et al. also demonstrated a synergistic effect on endothelial cell proliferation upon co-stimulation by FGF and VEGF [49]. In addition, it has been reported that FGF and VEGF have significantly greater effects in combination, compared to their individual effects in angiogenesis in vivo [50]. Specifically, the systolic pressure ratio of ischemic limb to healthy limb, the stem artery diameter, as well as the capillary density of New Zealand White rabbits treated with FGF and VEGF in combination were significantly greater than FGF or VEGF treated alone [50]. These results are consistent with the model prediction that R(pERK) is greater than one, where pERK is expected to directly influence proliferation. Moreover, Ratajska et al. showed that co-stimulation with FGF and VEGF did not have synergistic effect on migration distance in E12 embryonic hearts [51], which is consistent with our model prediction that R(pAkt) is less than one for all combinations of FGF and VEGF simulated, assuming pAkt directly influences migration. The model predictions for the pERK and pAkt responses following stimulation by FGF and VEGF mirrors these experimental observations, providing confidence in the model and its utility.

The molecular-detailed model presented here can be applied in various ways. We can use the model to increase understanding of the FGF- and VEGF-mediated angiogenic mechanisms and provide quantitative insight regarding the downstream signaling that mediates a cell’s response. As such, our work complements models that predict cellular behavior. Norton and Popel constructed a computational model to study vessel growth in tumor and showed that the proliferation rate has a greater effect on the spread and extent of vascular growth compared to the migration rate [52]. The simulations from our model are in line with their results, as pERK is more responsive to changing the ligand concentration (from 0.01 nM to the saturation concentration) compared to pAkt. And our model provides a detailed mechanistic explanation regarding their model predictions. Thus, the model can be utilized in combination with other modeling frameworks that predict cellular behaviors but do not yet take intracellular signaling into account [53, 54].

This model can also be used to study the efficiency of pro- or anti-angiogenic therapies. Currently, there are inhibitors targeting the ERK and Akt signaling networks, such as LY294002 and wortmannin (PI3K inhibitors), and PD98059 (MEK inhibitor) [55]. These inhibitors reduce pAkt and pERK levels [55] and further inhibit endothelial migration [56, 57] and proliferation [58], respectively. These inhibitors also reduce overall tube formation [55]. Interestingly, Hoeflich and coworkers showed that the MEK inhibitor PD0325901 upregulates the PI3K pathway signaling [59]. Our model is consistent with this observation and shows that inhibiting MEK significantly reduces pERK induced by FGF or the combination of FGF and VEGF, but actually increases the pAkt response (Fig. 7). The model predicts other instances where targeting certain parameters leads to opposing effects on pERK and pAkt. For example, decreasing the PI3K level and the rate of VEGFR2-induced PI3K phosphorylation or increasing Ras-GDP level can inhibit VEGF-induced pAkt, but promote VEGF-induced pERK (Fig. 7c, d). Overall, our model can predict the important variables that influence pERK and pAkt and how the concentrations of these signaling species are affected. These predictions can supplement experimental studies and provide insight into investigating the efficiency of targeting particular variables as pro- or anti-angiogenic strategies.

We acknowledge some limitations in our model. Firstly, some assumptions were made during model construction. We simplified certain reactions that occur upstream of activating MEK/ERK and PI3K/Akt pathways in order to focus on the effects of FGF and VEGF and their interactions. It has been reported that the PLC *γ* activation via VEGFR2 and FGFR further leads to PKC activation [9, 60]. However, the molecular detail relating PKC to ERK signaling is not clear. For example, some studies show that PKC may activate Ras and trigger Raf-MEK-ERK signaling [61–63], while PKC has been showed to activate ERK via Raf [64–66]. Another study reported that PKCα may activate MEK, independently of Raf and Ras, to further activate ERK signaling [67]. Therefore, we simplified certain reactions, and we can incorporate this detail when the protein-protein interactions in this pathway becomes available. Also, we excluded VEGFR1 and neuropilin-1 (NRP1) since VEGFR2 is thought to be the main receptor on endothelial cells [68]. While it has been shown that VEGFR1 promotes signal transduction [69], it is largely considered to be a decoy receptor [70]. In addition, NPR1 primarily acts as a coreceptor for VEGFR1 and VEGFR2 [68]. We can incorporate the contributions of VEGFR1 and NRP1 into the model in future studies. If these VEGF binding molecules were included, the effective concentration of VEGF would be lower and thus the magnitude of VEGF-induced certain responses may change. Moreover, we modeled FGF-mediated activation of Akt via FRS2 in the same way that VEGF promotes Akt activation through VEGFR2, and we used the VEGF kinetic parameters as a starting guess for the parameter fitting. This is because the FRS2-mediated protein-protein interactions that promote Akt signaling are not fully known, and there is a scarcity of quantitative data for the kinetics rates of FGF-induced PI3K activation. It has been reported that phosphorylated Akt deactivates Raf [71, 72]; however, experimental and computational studies have shown that MAPK and PI3K/Akt pathways act independently for a number of different cell types [73, 74]. Since there is lack of quantitative data for the kinetic parameters for Akt-mediated deactivation of Raf, and those parameters were not shown to influence the main model outputs (their *Sti* values are less than 0.22, see detail in Sensitivity analysis section in Methods), we did not include this feedback in our model. In the future, we can implement this interaction as more detailed mechanistic information becomes available. Finally, we only studied the pERK and pAkt responses over 2 hours in order to understand the initial effects of FGF and VEGF stimulation. Future work can expand our model to predict the downstream effects of this initial signaling, which occur on a longer timescale. However, despite these limitations, our model provides novel insights into angiogenic signaling, complements experimental studies, and is a platform for a range of future investigations.

## Conclusion

In conclusion, we developed a mathematical model to characterize the dynamics of pERK and pAkt following stimulation with two main pro-angiogenic factors, FGF and VEGF. The model quantitatively studies particular aspects of FGF and VEGF interactions network in ERK and Akt phosphorylation, provides mechanistic insight into their signaling network, and identifies specific potential angiogenic targets that can be altered to modulate ERK and Akt activation. The model provides a molecularly detailed understanding of the regulation of endothelial cell angiogenesis signaling in terms of ERK and Akt activation upon stimulation with FGF and VEGF. Thus, our work can aid in the development of pro- and anti-angiogenic strategies that particularly target ERK and/or Akt responses induced by FGF and VEGF.

## Supplementary information

**Additional file 1: Table S1.** List of model reactions. **Table S2.** List of species with non-zero initial concentrations. **Table S3.** List of model parameters. **Table S4.** The total sensitivity index Sti values. **Table S5.** Fitted initial concentrations and parameters with adjusted bounds.

**Additional file 2: Supplementary Figures and Legends.**

**Additional file 3: Mathematical model.** MATLAB.m file containing model code.

## Data Availability

All data generated or analyzed during this study are included in this published article and its supplementary information files.

## Abbreviations

Akt: Protein kinase B
eFAST: Extended Fourier amplitude sensitivity test
ERK: Extracellular regulated kinase
FGF: Fibroblast growth factor
FGFR1: Fibroblast growth factor receptor 1
FRS2: Fibroblast growth factor receptor substrate 2
GDP: Guanosine diphosphate
HSGAG: Heparan sulfate glycosaminoglycan
HUVECs: Human umbilical vein cells
MAPK: Mitogen-activated protein kinase
MEK: Mitogen-activated protein kinase kinase
pAkt: Phosphorylated protein kinase B
PDGF: Platelet-derived growth factor
pERK: Phosphorylated extracellular regulated kinase
PIP2: Phosphatidylinositol 4,5-bisphosphate
PIP3: Phosphatidylinositol (3,4,5)-trisphosphate
pMEK: Phosphorylated mitogen-activated protein kinase kinase
PP2A: Protein phosphatase 2
ppAkt: Doubly phosphorylated protein kinase B
ppERK: Doubly phosphorylated extracellular regulated kinase
ppMEK: Doubly phosphorylated mitogen-activated protein kinase kinase
pR2: Phosphorylated vascular endothelial growth factor receptor 2
PSO: Particle Swarm Optimization
Ptase: Phosphatase
PTEN: Phosphatase and tensin homolog gene
TSP1: Thrombospondin-1
VEGF: Vascular endothelial growth factor
VEGFR2: Vascular endothelial growth factor receptor 2
