# Supplementary material for: ERK and Akt exhibit distinct signaling responses following stimulation by pro-angiogenic factors
Source: Cell Commun Signal. 2020 Jul 17;18:114. doi: 10.1186/s12964-020-00595-w (PMC7368799; doi:10.1186/s12964-020-00595-w)
Supplement: Supplementary file 3 — Additional file 2: Supplementary Figures and Legends. [file 12964_2020_595_MOESM3_ESM.pdf]

ERK and Akt exhibit distinct signaling responses following stimulation by pro-angiogenic factors

Min Song and Stacey D. Finley

Supplementary Figures

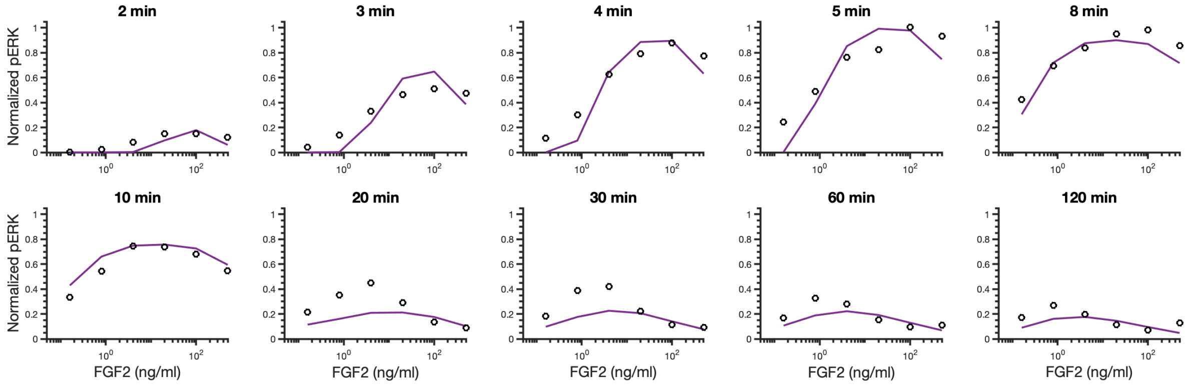

Figure S1. Dose response of pERK for FGF stimulation. The circles are NCI-H1730 experimental data. Curves are the mean values of the 15 best fits. Shaded regions show standard deviation of the fits.

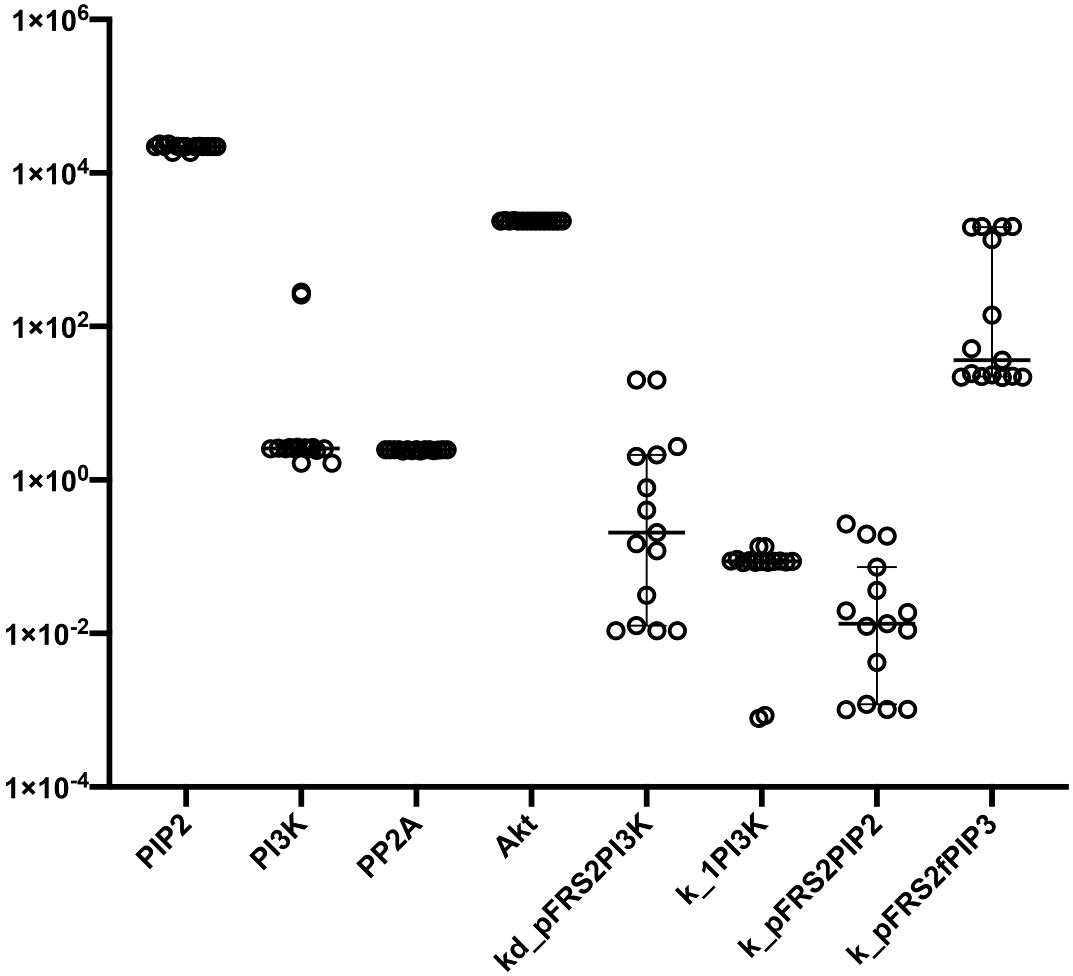

Figure S2. Model values estimated in fitting. (A) Distribution of fitted initial concentrations and model parameters. Each dot represents one fit. Bars are median ± 95% confidence interval. Concentrations in nM.

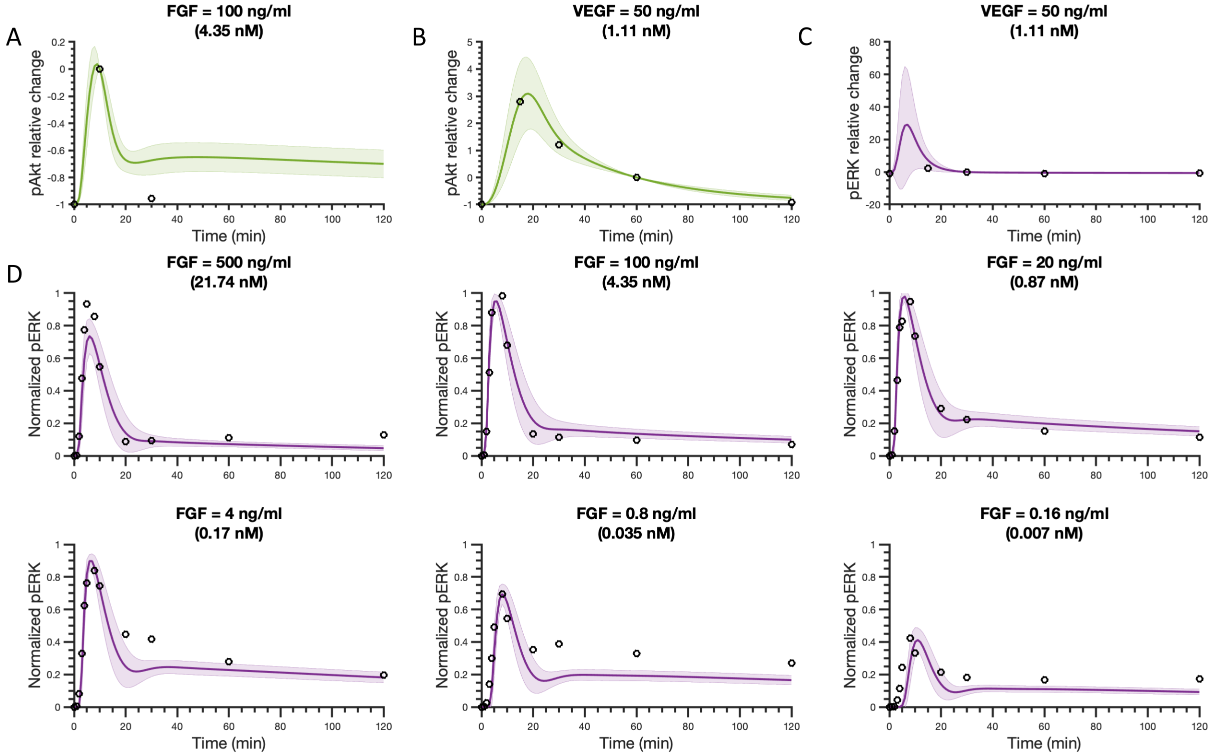

Figure S3. Monte Carlo simulations for training data for FGF or VEGF stimulation. (A) Relative change of pAkt for 100 ng/ml (4.35 nM) FGF stimulation. (B) Relative change of Akt phosphorylation upon stimulation with 50 ng/ml (1.11 nM) VEGF. (C) Relative change of ERK phosphorylation following stimulation with 50 ng/ml (1.11 nM) VEGF. (D) Normalized pERK dynamics in response to FGF concentrations ranging from 0.16 to 500 ng/ml (0.007 – 21.74 nM). Circles in Panels A-C are HUVEC experimental data and circles in Panel D are NCI-H1730 experimental data. Curves are the mean values of the 1,000 Monte Carlo simulations. Shaded regions show standard deviation.

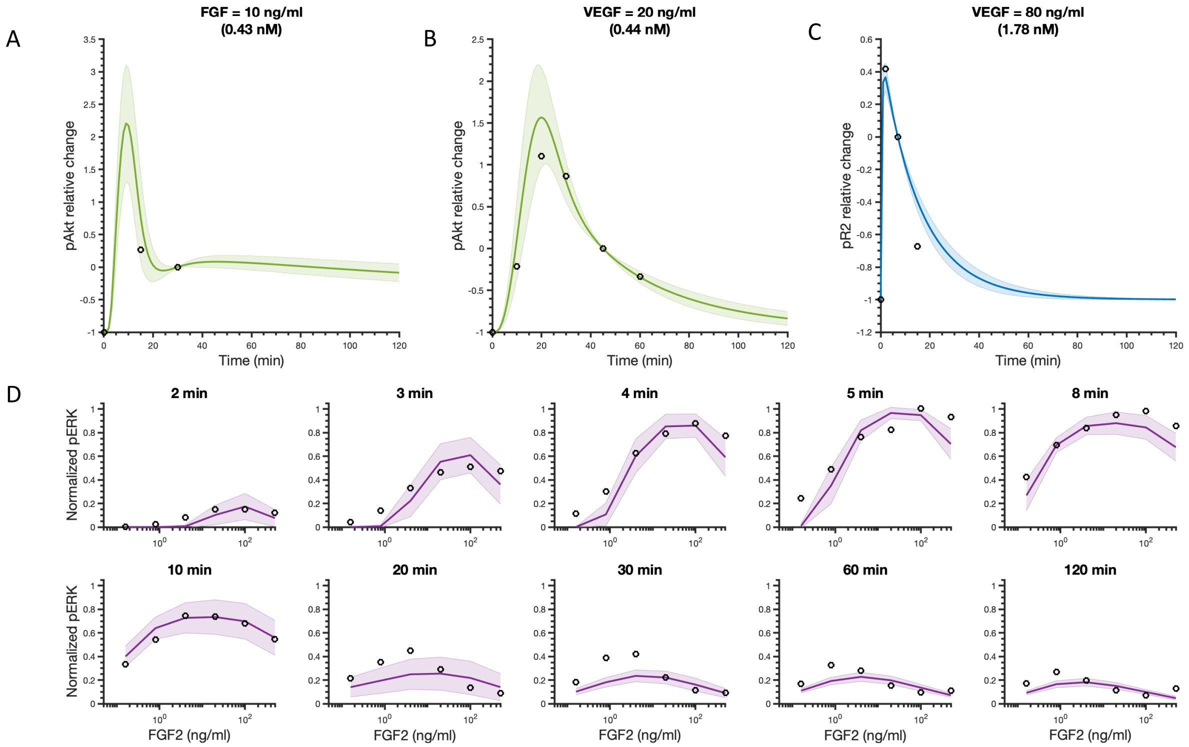

Figure S4. Monte Carlo simulations for model validation data for FGF or VEGF stimulation. (A) Relative change of pAkt by the stimulation with 10 ng/ml (0.43 nM) FGF. (B) Relative change of Akt phosphorylation upon stimulation with 20 ng/ml (0.44 nM) VEGF. (C) Relative change of VEGFR2 phosphorylation upon stimulation with 80 ng/ml (1.78 nM) VEGF. (D) Dose response of pERK for FGF stimulation. Circles in Panels A-C are experimental data from HUVECs, and circles in Panel D are experimental data from the NCI-H1730 cell line. Curves are the mean values of the 1,000 Monte Carlo simulations. Shaded regions show standard deviation.

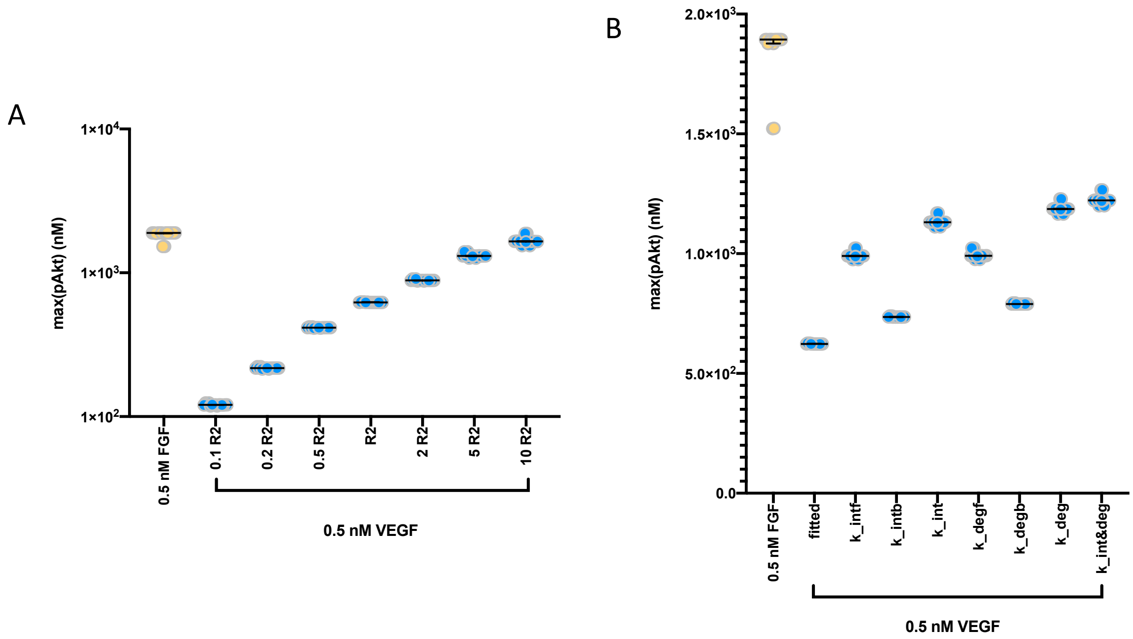

Figure S5. Effect of varying VEGFR2 initial concentrations and relevant parameters on pAkt response. Maximum pAkt induced by 0.5 nM FGF or 0.5 nM VEGF alone with varied initial VEGFR2 concentrations (A) and decreased VEGFR2 related parameters (B). We decreased VEGFR2 internalization and degradation rates individually and together to be the same as the corresponding FGFR kinetic parameters. Yellow: FGF; Blue: VEGF. Each dot represents one fit. Bars are median ± 95% confidence interval.

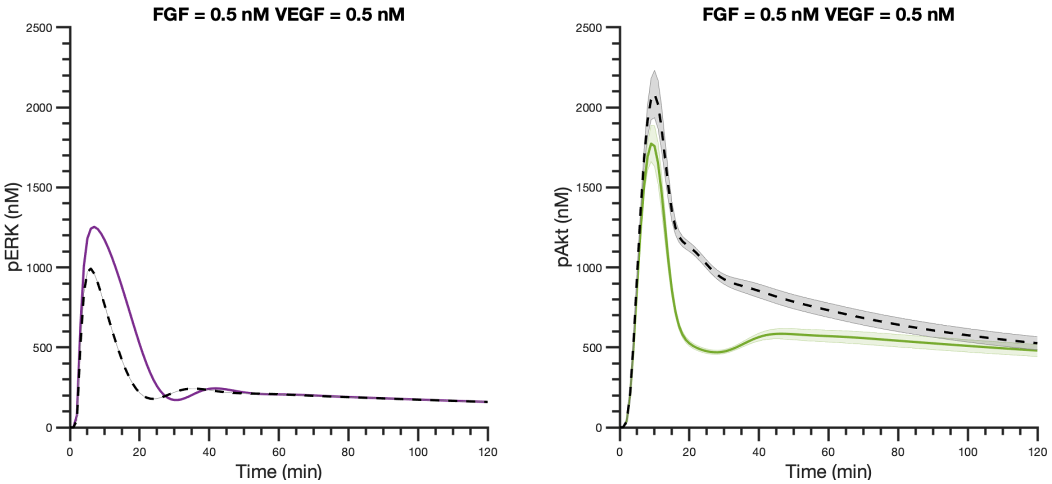

Figure S6. Comparison of predicted maximum pERK (left) and pAkt (right) responses with co-stimulation (solid lines) and the summation of mono-stimulation (dashed lines) by 0.5 nM FGF and 0.5 nM VEGF.

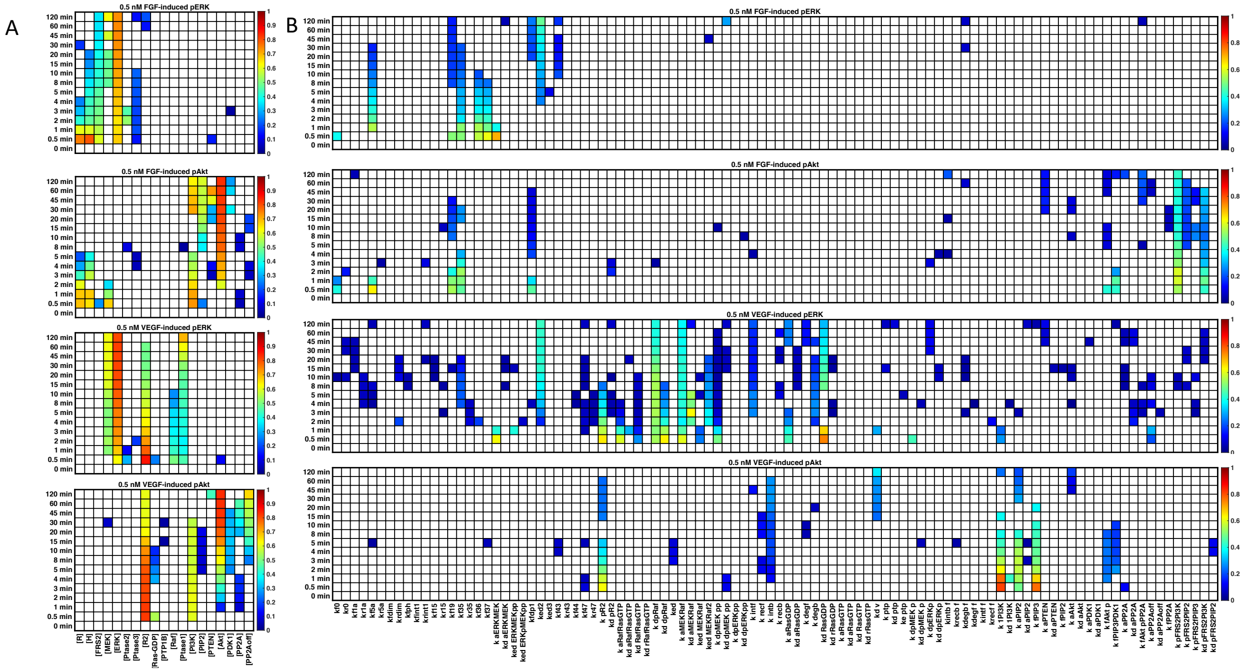

Figure S7. Representative results of eFAST sensitivity analysis. Based on the behaviors of maximum pERK and pAkt that reach a plateau as the FGF and/or VEGF concentration increases, we selected five representative concentrations to capture the low (0.01 nM and 0.05 nM), intermediate (0.1 nM), and high (0.5 nM and 1 nM) levels of responses. Here shows the total sensitivity index (*Sti*) values for (A) Initial concentrations and (B) Kinetic parameters quantify how these inputs influence pERK and pAkt levels upon stimulation by 0.5 nM FGF and 0.5 nM VEGF. The color bars indicate the *Sti* values.
